# Incidence and risk factors for acute kidney injury in head and neck cancer patients treated with concurrent chemoradiation with high-dose cisplatin

**DOI:** 10.1186/s12885-019-6233-9

**Published:** 2019-11-08

**Authors:** Maurice J. D. L. van der Vorst, Elisabeth C. W. Neefjes, Elisa C. Toffoli, Jolanda E. W. Oosterling-Jansen, Marije R. Vergeer, C. René Leemans, Menno P. Kooistra, Jens Voortman, Henk M. W. Verheul

**Affiliations:** 1 Department of Medical Oncology, Cancer Center Amsterdam, VU University Medical Center, De Boelelaan 1117,Rm 3A46, Amsterdam, 1081HV The Netherlands; 2 Department of Internal Medicine, Rijnstate Hospital, Arnhem, the Netherlands; 3 Department of Radiation Oncology, Cancer Center Amsterdam, Amsterdam UMC, Vrije Universiteit, Amsterdam, the Netherlands; 4 Department of Otolaryngology-Head and Neck Surgery, Cancer Center Amsterdam, Amsterdam UMC, Vrije Universiteit, Amsterdam, the Netherlands

**Keywords:** Locally advanced squamous cell carcinoma of the head and neck, High-dose cisplatin, Chemoradiation, Acute kidney injury, Risk factors

## Abstract

**Background:**

Three-weekly high-dose cisplatin (100 mg/m^2^) is considered the standard systemic regimen given concurrently with postoperative or definitive radiotherapy in locally advanced squamous cell carcinoma of the head and neck (LA-SCCHN). Concurrent chemoradiation (CRT) with high-dose cisplatin is associated with significant acute and late toxicities, including acute kidney injury (AKI). The aims of this study were to investigate the incidence of AKI in patients with LA-SCCHN during and after treatment with high-dose cisplatin-based CRT, to identify risk factors for cisplatin-induced AKI, and to describe the impact of AKI on long-term renal function and treatment outcomes.

**Methods:**

This is a retrospective cohort study with measurements of renal function before CRT, weekly during CRT, every 1 or 2 days during hospitalizations, and 3 and 12 months after CRT in patients with LA-SCCHN. AKI was defined as increase in serum creatinine (sCr) of ≥1.5 times baseline or by ≥0.3 mg/dL (≥26.5 μmol/L) using the Kidney Disease Improving Global Outcomes (KDIGO) classification. Logistic regression models were estimated to analyze renal function over time and to identify predictors for AKI.

**Results:**

One hundred twenty-four patients completed all measurements. AKI was reported in 85 patients (69%) with 112 episodes of AKI. Sixty of 85 patients experienced 1 AKI episode; 20 patients experienced ≥2 AKI episodes. Ninety-three (83%) AKI episodes were stage 1, 13 (12%) were stage 2, and 6 (5%) AKI episodes were stage 3. Median follow-up time was 29 months (Interquartile Range, IQR 22–33). Hypertension (Odds Ratio, OR 2.7, 95% Confidence Interval, CI 1.1–6.6; *p* = 0.03), and chemotherapy-induced nausea and vomiting (CINV; OR 4.3, 95% CI 1.6–11.3; *p* = 0.003) were associated with AKI. In patients with AKI, renal function was significantly more impaired at 3 and 12 months post-treatment compared to patients without AKI. AKI did not have a negative impact on treatment outcomes.

**Conclusion:**

AKI occurred in 69% of patients with LA-SCCHN undergoing CRT with high-dose cisplatin. Long-term renal function was significantly more impaired in patients with AKI. Hypertension and CINV are significant risk factors. Optimizing prevention strategies for CINV are urgently needed.

## Background

Three-weekly high-dose cisplatin (100 mg/m^2^) is considered the standard systemic regimen given concurrently with postoperative or definitive radiotherapy in locally advanced squamous cell carcinoma of the head and neck (LA-SCCHN) [1–3]. The additional absolute benefit in overall survival of adding cisplatin chemotherapy has been best estimated as 6.5% at 5 years when compared with radiotherapy alone [4]. However, concurrent high-dose cisplatin is associated with significant acute and late toxicities [5, 6]. Acute kidney injury (AKI) is a common and serious side effect of high-dose cisplatin-based concurrent chemoradiation (CRT). AKI is a predictor of immediate and long-term adverse outcomes. Even a minor acute reduction in kidney function has an adverse prognosis [7].

The incidence of cisplatin-induced AKI has been reported before [5, 8–10]. However, development of AKI during high-dose cisplatin-based CRT is underreported using the Kidney Disease Improving Global Outcomes (KDIGO) criteria [11], which are the most recent and preferred criteria for diagnosis and staging of AKI. Also, little is known about the impact of AKI on long-term renal function and treatment outcomes in patients with LA-SCCHN. Early detection of AKI enables early intervention, which might lessen treatment burden and improves efficacy and cost-effectiveness of care [12]. Therefore, it is clinically relevant to identify potentially modifiable risk factors for cisplatin-induced AKI in this patient group.

The purpose of this study is to answer the following questions: (1) what is the incidence of AKI during treatment with high-dose cisplatin-based CRT for LA-SCCHN according to KDIGO criteria, (2) which predictors for development of cisplatin-induced AKI can be identified, and (3) what are the long-term consequences of cisplatin-induced AKI in this patient group?

## Methods

### Study design

From January 2017 to July 2017, patient data were collected retrospectively by two investigators (M.V. and E.N.) from electronic medical records (EMRs) between January 2011 (introduction of EMRs in our center) and January 2014.

### Patient population

Patients, both female and male, 18 years or older, with histologically proven, resectable high-risk or not-resectable LA-SCCHN, who were treated with three-weekly high-dose (100 mg/m^2^) cisplatin-based CRT from January 2011 to January 2014 at the Amsterdam University Medical Center, VU University, were included in this study. Exclusion criteria were a history of AKI or a creatinine clearance of ≤60 mL/min/1.73 m^2^ (estimated by the Cockcroft-Gault equation) before start of CRT. Other exclusion criteria were diagnosis of nasopharyngeal carcinoma, previous treatment with radiotherapy and/or chemotherapy, and treatment with biologicals. This retrospective study was not subject to the Dutch Medical Research Involving Human Subjects (WMO) act as was determined by the Medical Ethics Committee of the Amsterdam UMC, Vrije Universiteit Amsterdam.

### Chemotherapy

Cisplatin (100 mg/m^2^) was administered intravenously on day 1 of a three-weekly cycle for a total of 3 courses, with pre-hydration containing 2000 mg magnesium sulfate and 20 milliequivalents per Liter (mEq/L) of potassium chloride in 1000 mL of 0.9% normal saline over a 2-h period, and post-hydration containing 2000 mg magnesium sulfate and 20 mEq/L of potassium chloride in 4000 mL of 0.9% normal saline over a 20-h period.

Prophylactic antiemetic therapy to prevent chemotherapy-induced nausea and vomiting (CINV) was prescribed according to international guidelines [13, 14], containing a three-drug regimen, which included dexamethasone, the serotonin receptor antagonist (5-HT_3_ RA) ondansetron, and the neurokinin-1 receptor antagonist (NK_1_ RA) aprepitant intravenously before administration of cisplatin (day 1), followed by aprepitant on days 2 and 3, and dexamethasone on days 2 to 4 taken orally. The use of rescue antiemetics was allowed and reported in the EMR.

### Measurements

Demographic and tumor characteristics, tumor and nodal stage (7th edition of the American Joint Committee on Cancer **(**AJCC**)** TNM classification of malignant tumors), medical history, weight and height, age-adjusted Charlson Comorbidity Index (CCI) [15], and Eastern Cooperative Oncology Group (ECOG) performance status score were derived from the EMRs of the included patients. Information on the use of potentially nephrotoxic co-medications was obtained by medical prescription history from the week before start of treatment until the last day of chemoradiation. The drugs documented included all categories of diuretics, angiotensin-converting-enzyme inhibitors, angiotensin II receptor blockers, non-steroidal anti-inflammatory drugs (NSAIDs), proton-pump inhibitors, lithium, haloperidol, and intravenous contrast media. Data on early termination of cisplatin or dose reductions, radiotherapy delay or truncations, occurrence of CINV, the use of rescue antiemetics, and the number and length of emergency hospitalizations were also obtained, including the reason for treatment modifications and emergency admissions.

Serum creatinine (sCr) values were derived from the clinical laboratory database at baseline (day before start CRT), weekly during CRT, at least every other day during (emergency) hospitalizations, and 3 and 12 months after completion of CRT. The criteria for AKI based on the KDIGO criteria were applied [11]. AKI (stage 1) was defined by sCr rise of greater than or equal to 26.5 μmol/l within 48 h, or sCr increase greater than or equal to 1.5-fold from the baseline reference value. Stage 2 AKI was defined as a greater than or equal to 2.0- to 2.9 fold increase from baseline reference sCr. Stage 3 AKI was defined as a greater than or equal to threefold increase from baseline reference sCr, or increase of 354 μmol/l, or commenced on renal-replacement therapy irrespective of stage of AKI. The reference sCr is defined as the lowest creatinine value recorded within 3 months of the event, or from repeat sCr within 24 h, or estimated from the nadir sCr value if a patient recovers from AKI. The urine output criterion was not used in this study. Disease free survival (DFS) and disease-specific mortality (DSM) were assessed from the last day of radiotherapy until disease recurrence or death, respectively.

### Statistics

Descriptive analyses were used to describe patient and treatment characteristics and the incidence of AKI. To indicate predictors for cisplatin-induced AKI, univariate analysis was used to analyze the association between AKI and age (< 60 years vs, ≥60 years), sex, ECOG performance status score before start of treatment (< 2 vs. ≥2), presence of hypertension (defined as systolic pressure > 140 mm Hg (mmHg) or diastolic pressure > 90 mmHg) before start of treatment (yes vs. no), presence of diabetes mellitus (yes vs. no), presence of cognitive impairment (yes vs. no), number of nephrotoxic co-medications taken in the week before start of CRT (< 2 vs. ≥2), number of pack-years (< 10 years vs. ≥ 10 years), excessive alcohol consumption (< 14 units per week vs. ≥ 14 units per week), primary LA-SCCHN tumor site (oropharyngeal vs. non-oropharyngeal), and occurrence of clinically relevant CINV (defined as administration of rescue antiemetics and/or hospital admission to provide targeted care for CINV) during treatment. Variables in the univariate logistic regression analysis with an association *p* < 0.20 were included as independent variables into the multivariate logistic regression model. In the multivariate analysis model, *p* values < 0.05 were considered statistically significant.

The paired samples *t* test was used to compare mean SCr values at baseline, and at 3 and 12 months post-treatment, in both patients with AKI during treatment, and those without (non-AKI patients). The independent samples *t* test was used to compare the means of SCr values between AKI and non-AKI patients at baseline, and at 3 and 12 months post-treatment. Kaplan-Meier and log-rank methods were used to compare the curves of DFS and DSM between AKI and non-AKI patients.

Analyses were performed with IBM SPSS statistics version 22 (Chicago, IL, United States).

## Results

A total of 124 patients were included in this study. The median age was 60 years (range, 30 to 74 years), 78% of patients were male, and 94% had ECOG performance status 0 to 1 (Table 1). Twenty percent of patients had hypertension, age-adjusted CCI score was 0 to 1 in 74% of patients. Most patients (74%) had a smoking history of ≥10 pack-years, and 20% indicated excessive use of alcohol. Median number of potentially nephrotoxic co-medications was 2 (range, 0 to 3). Primary LA-SCCHN tumor site was the oral cavity or oropharynx in 71% of patients, and the hypopharynx in 12%. Fifty-six percent of patients had T3 or T4 LA-SCCHN, and 85% had node-positive disease. Mean sCr value was 66 μmol/l (Standard Deviation, SD 12). Eighty-five patients (69%) were re-admitted at least once for AKI during CRT.

**Table 1:** Baseline Patient and Tumor Characteristics

Characteristic	Total (*N* = 124)	AKI (*n* = 85)	Non-AKI (*n* = 39)
Median age, (range), years	60 (30–74)	60 (30–71)	59 (41–74)
Male	97 (78)	67 (79)	30 (77)
ECOG performance status			
0	41 (33)	26 (31)	15 (38)
1	76 (61)	55 (65)	21 (54)
2	6 (5)	3 (4)	3 (8)
Not specified	1 (1)	1 (1)	
Hypertension	25 (20)	20 (24)	5 (13)
Diabetes mellitus	9 (7)	7 (8)	2 (5)
Cognitive impairment	8 (6)	4 (5)	4 (10)
CCI			
0–1	92 (74)	61 (72)	31 (79)
2–3	32 (26)	24 (28)	8 (21)
Smoking			
≥ 10 pack-years	92 (74)	65 (76)	27 (69)
Alcohol			
≥ 14 Units/week	46 (37)	35 (41)	11 (28)
Number of nephrotoxic co-medications, median (range)	2 (0–3)	2 (0–3)	2 (1–3)
Mean SCr (SD), μmol/l	66 (12)	66 (12)	65 (12)
Primary site			
Oral cavity / oropharynx	88 (71)	58 (68)	30 (77)
Hypopharynx	15 (12)	12 (14)	3 (8)
Larynx	17 (14)	12 (14)	5 (13)
Other	4 (3)	3 (4)	1 (3)
Tumor stage			
T1–2	46 (37)	29 (34)	17 (44)
T3–4	69 (56)	51 (60)	18 (46)
Unknown	6 (5)	5 (6)	1 (3)
Nodal stage			
N0	15 (12)	12 (14)	3 (8)
N+	106 (85)	71 (84)	35 (90)
Unknown	3 (2)	2 (2)	1 (3)

Data given as No. (%), unless otherwise noted

*Abbreviations*: *ECOG* Eastern Cooperative Oncology Group Performance Status Score (WHO), *CCI* Age-adjusted Charlson Comorbidity Index, *SCr* Serum creatinine (μmol/L), *SD* standard deviation

**Fig. 1:**
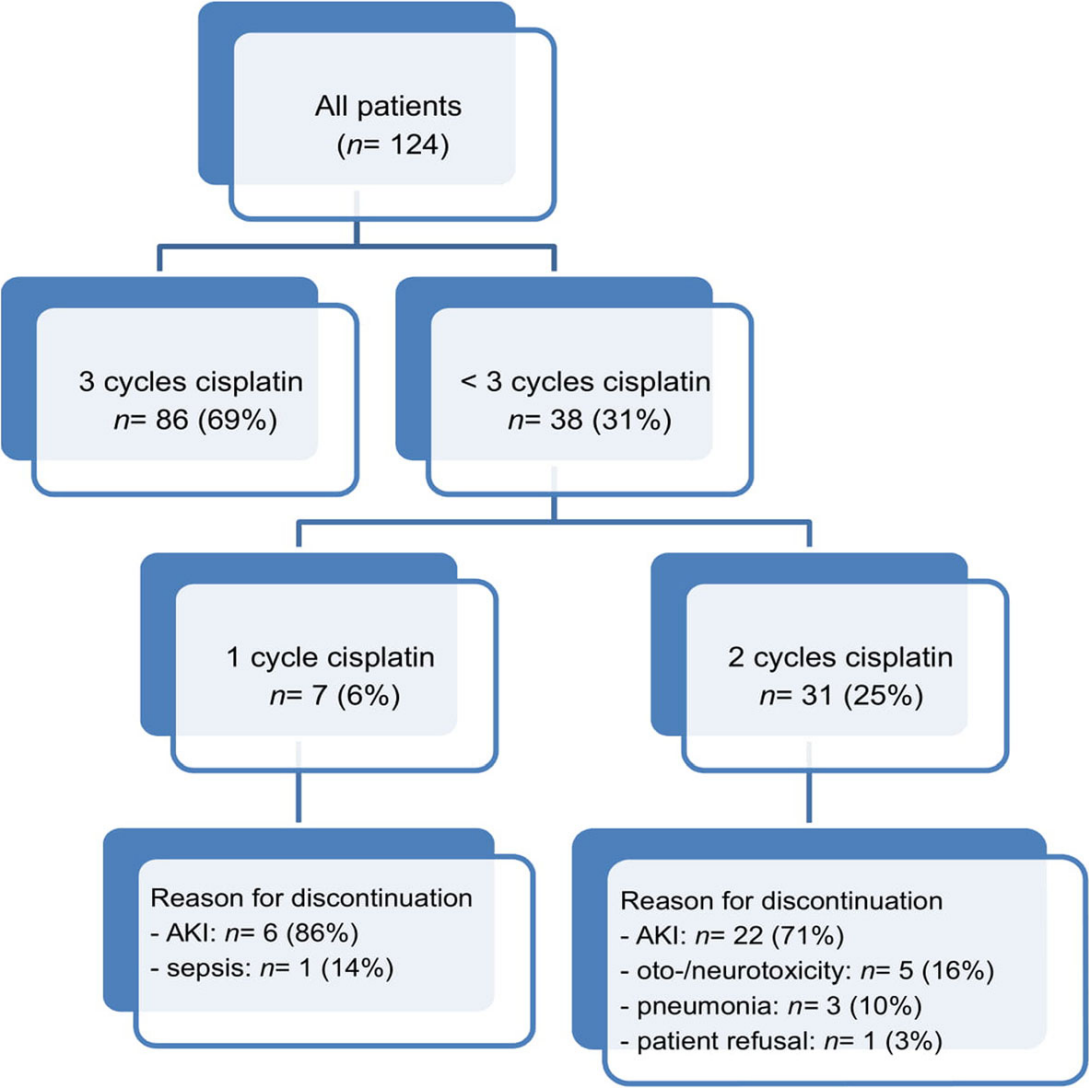
Patient Disposition

AKI was reported in 85 patients (69%) with 112 episodes of AKI. Sixty of 85 patients (71%) experienced 1 AKI episode; 20 patients (29%) experienced ≥2 AKI episodes. Ninety-three (83%) AKI episodes were stage 1, 13 (12%) were stage 2, and 6 (5%) AKI episodes were stage 3. Eighty-six patients (69%) received all 3 preplanned courses of cisplatin (cumulative dose 300 mg/m^2^) without dose adjustment (Fig. 1). Thirty-eight patients (31%) prematurely discontinued cisplatin treatment; 7 patients after the first cycle, and 31 patients after 2 cycles of cisplatin. Reasons for discontinuation was AKI in 28 patients (74%) and infection/sepsis in 4 patients (11%). Median cumulative dose of cisplatin was 259 mg/m^2^ (86% of preplanned dose) in the AKI group and 269 mg/m^2^ (90% of preplanned dose) in the non-AKI group (*p* = 0.36). All patients but 2 (sepsis, *n* = 1; patient refusal, *n* = 1) received the preplanned, scheduled radiotherapy dose.

### Predictors for cisplatin-induced AKI

Hypertension, ≥2 nephrotoxic co-medications, excessive alcohol consumption, and CINV were variables in the univariate analysis with an association *p* < 0.20 with cisplatin-induced AKI (Table 2). The multivariate logistic regression model shows that hypertension (Odds Ratio (OR) 2.7, 95% Confidence Interval (CI) 1.1–6.6; *p* = 0.03), and CINV (OR 4.3, 95% CI 1.6–11.3; *p* = 0.003) were significantly associated with cisplatin-induced AKI.

**Table 2:** Univariate and Multivariate Logistic Regression for AKI (KDIGO)

Variables	OR (95% CI)	Univariate *p* value	OR (95% CI)	Multivariate *p* value
Age, ≥60 years	1.1 (0.5–2.3)	0.85		
Male gender	0.9 (0.4–2.2)	0.81		
ECOG performance status, ≥2	0.4 (0.1–2.3)	0.34		
Hypertension, yes	2.1 (0.7–6.1)	**0.17**	2.7 (1.1–6.6)	**0.03**
Diabetes, yes	1.7 (0.3–8.4)	0.54		
Cognitive impairment, yes	0.4 (0.1–1.8)	0.25		
CCI, ≥ 2	1.5 (0.5–4.1)	0.45		
Number of nephrotoxic co-medications, ≥ 2	2.1 (0.8–5.4)	**0.12**	1.9 (0.7–5.2)	0.20
Smoking, ≥10 pack-years	1.4 (0.6–3.4)	0.39		
Alcohol, ≥14 U/week	1.8 (0.8–4.0)	**0.17**	2.3 (0.7–7.0)	0.15
Primary tumor site, not oropharynx	0.6 (0.3–1.5)	0.32		
CRINV, yes	3.0 (1.2–7.3)	0.02	4.3 (1.6–11.3)	**0.003**

Note: Bold values in the univariate logistic regression model indicate *p*-values <0.20 as criterion for selection and entry into the multivariate analysis. Significant *p*-values in the multivariate analysis model (<0.05) are also denoted in bold.

*Abbreviations*: *KDIGO* kidney disease improving global outcomes definition and staging system, *OR* odds ratio, *CI* confidence interval, *ECOG* Eastern Cooperative Oncology Group Performance Status Score, *CCI* Age-adjusted Charlson Comorbidity Index, *CINV* chemoradiation-induced nausea and vomiting

### Long-term renal function and treatment outcomes

Data on sCr were available for all patients at baseline, for 108 patients (87%) at 3 months, and for 82 patients (66%) at 12 months post-treatment. There were no significant differences at baseline; mean sCr was 66 μmol/L (SD 12) for AKI patients, and 65 μmol/L (SD 12) for non-AKI patients (*p* = 0.78). At 3 months (Table 3), compared to baseline values, renal function was impaired in AKI patients (mean sCr 103 μmol/L, SD 36; *p* = 0.001), and also in non-AKI patients (mean sCr 79 μmol/L, SD 14; *p* = 0.01). At 12 months, compared to baseline values, renal function was impaired in both AKI patients (mean sCr 100 μmol/L, SD 35; *p* = 0.002), and non-AKI patients (mean sCr 80 μmol/L, SD 21; *p* = 0.01). Compared to non-AKI patients, renal function was significantly more impaired in AKI patients at 3 months (*p* = 0.01) and at 12 months (*p* = 0.01).

**Table 3:** Renal Function

Renal function, mean sCr, μmol/l (SD)			
Time point	AKI	Non-AKI	*p* value^2^
Baseline	66 (12)	65 (12)	0.78
3 months	103 (36)	79 (14)	0.001
*p* value^1^	0.001	0.01	
12 months	100 (35)	80 (21)	0.01
*p* value^1^	0.002	0.01	

*Abbreviations*: *sCr* serum creatinine, *SD* standard deviation; *p*^*1*^, intra-group comparison of renal function to baseline sCr; *p*^*2*^, intergroup comparison of renal function

**Fig. 2:**
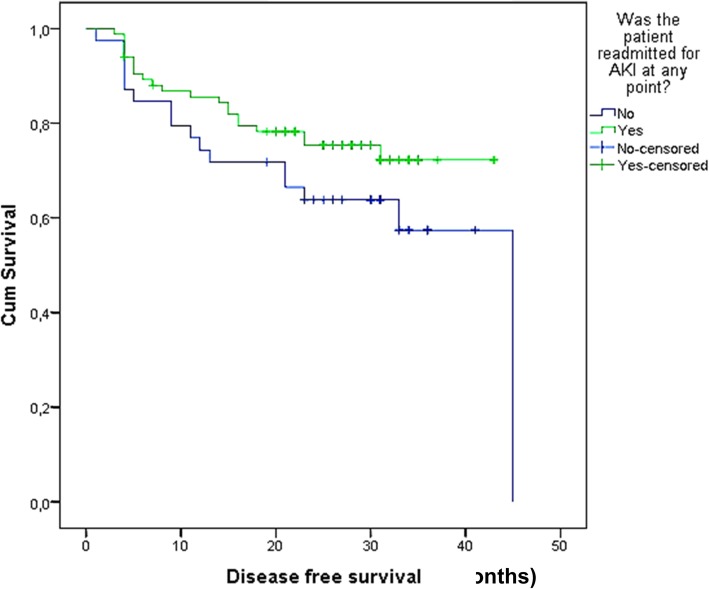
Disease Free Survival

**Fig. 3:**
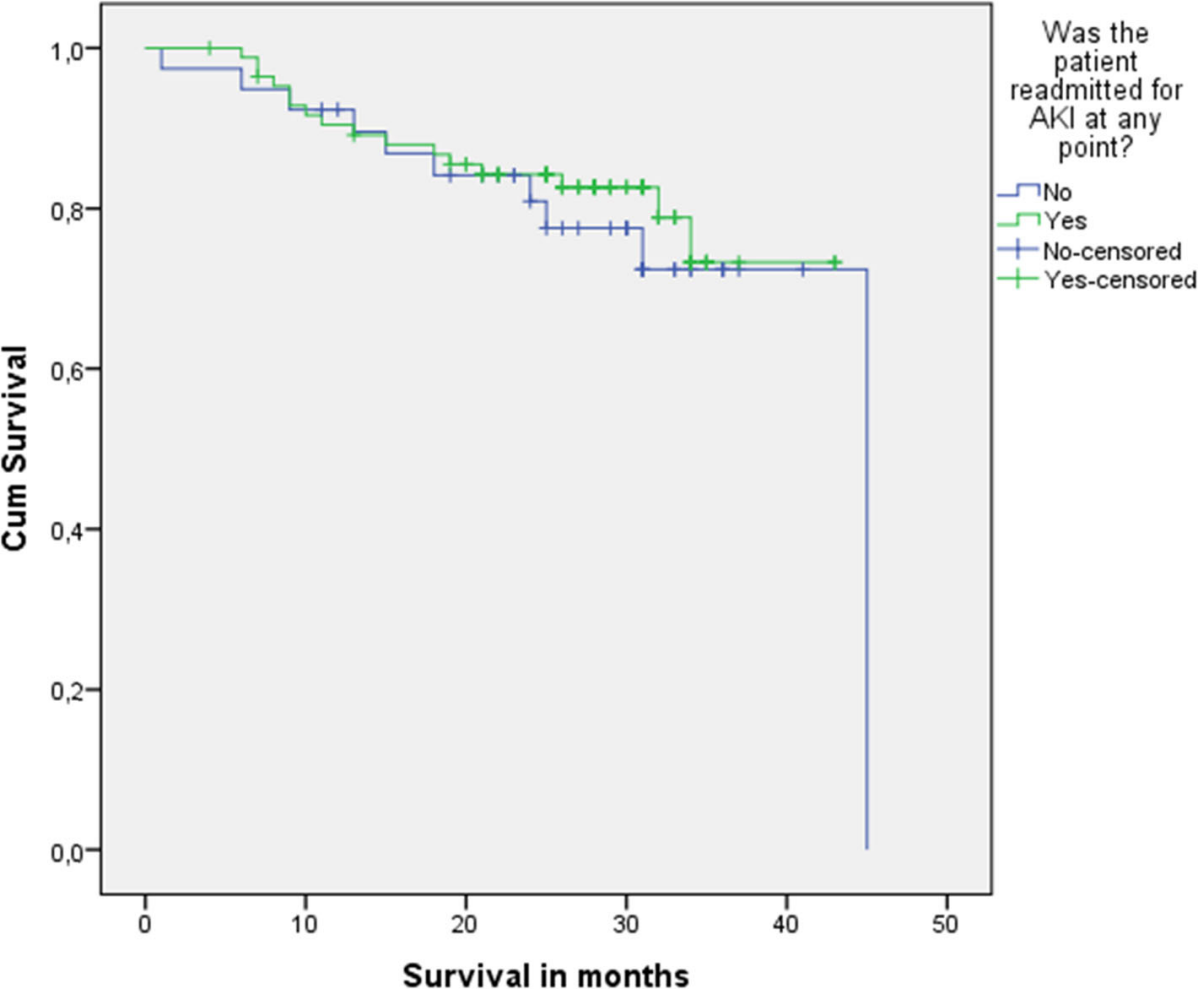
Disease-Specific Mortalit

Median follow-up time was 29 months (Interquartile Range, IQR 22–33) with no statistically significant difference between both groups. Disease recurrence rate was 25% in AKI patients, and 41% in non-AKI patients (OR 0.6, 95% CI 0.3–1.4; *p* = 0.22) (Fig. 2). DSM rate was 19% in AKI patients, and 26% in non-AKI patients (OR 1.8; 95% CI 0.2–14.9; *p* = 0.61) (Fig. 3).

## Discussion

The present retrospective cohort study shows that 69% of patients with LA-SCCHN developed AKI stage 1 or higher during treatment with high-dose cisplatin-based CRT, according to the KDIGO definition and staging criteria. Almost 30% of patients experienced 2 of more AKI episodes. The majority of AKI episodes (83%) was stage 1 according to KDIGO criteria; only 6% was AKI stage 3. Predictive risk factors for cisplatin-induced AKI included hypertension and uncontrolled CINV. Long-term impairment of renal function was observed in both AKI and non-AKI patients. However, renal function was significantly worse at 3 and 12 months in patients with AKI during CRT. DFS and DSM were comparable between AKI and non-AKI patients.

Cisplatin-induced AKI has been reported to occur in 1 to 46% of patients treated with high-dose cisplatin, depending on the described grade of nephrotoxicity and the used AKI definition and staging system [5, 8, 10, 16]. Previous studies often used the adverse events criteria for chemotherapy, Common Toxicity Criteria for Adverse Events (CTCAE). In early versions of CTCAE (version 2.0 and 3.0), grading of renal insufficiency was based solely on the x-fold increase of the sCr level with respect to the Upper Limits of Normal (ULN). CTCAE v2.0 and 3.0 have different cutoff values for renal insufficiency than KDIGO, and no provision of a time course, which complicate direct comparisons of AKI incidence and outcome. CTCAE version 4.0 (v4.0) was the first to define AKI as sCr exceeding 26.5 μmol/l. Cutoff values for AKI grade 1 to 3 in CTCAE v4.0 resemble those according to KDIGO. However, in contrast to KDIGO, there is no provision of a time course in CTCAE v4.0. In previous trials using CTCAE criteria, AKI grade 1 and 2 were seldomly reported; only AKI grade 3 (sCr > 3 x baseline or 354 μmol/l; hospitalization indicated) and grade 4 (life-threatening consequences; dialysis indicated) were reported. Consequently, the high incidence rate of AKI in the present study compared to previous studies is explained by the identification of low stage AKI by using the KDIGO system. KDIGO builds upon two earlier AKI classification systems: the Acute Kidney Injury Network (AKIN) and the Risk, Injury, Failure, Loss, End-Stage (RIFLE) criteria. Compared against AKIN and RIFLE, the incidence of AKI according to KDIGO is the highest due to the addition of an absolute increase criterion (≥0.3 mg/dl over 48 h) to the RIFLE definition and expansion of the time limit for percentage increase (≥ 50%) in the AKIN definition from 48 h to 7 days [17]. Therefore, AKI will be more frequently diagnosed at an early stage, if KDIGO is applied.

The standard approach to prevent cisplatin-induced nephrotoxicity is the administration of intravenous (iv) isotonic saline (≥3 L/day) to induced diuresis during cisplatin administration. However, the optimal hydration solution and regimen to prevent nephrotoxicity associated with cisplatin administration is unclear. There are no randomized trials that have compared different regimens and/or types of iv fluids. In this study, all patients received 5 L/day of iv isotonic saline according to protocol. Forced diuresis with mannitol is frequently used, although there is no evidence that this is required. There is also concern that mannitol may over-diurese some patients, resulting in dehydration [18]. Therefore, mannitol was not used in this study. There is insufficient evidence to support using furosemide for forced diuresis, unless there is evidence of fluid overload, as was applied in our study. Hypomagnesemia can upregulate OCT-2, leading to increased cisplatin transport to the kidneys, resulting in nephrotoxicity [19]. Several systematic reviews suggest that magnesium supplementation (8–16 milliequivalents [mEq]) may limit cisplatin-induced nephrotoxicity [18, 20]. In this study, 2000 mg (= 16 mEq magnesium) was administered to all patients according to protocol. Potassium supplementation was also included in the protocol. Several other approaches have been evaluated to prevent cisplatin-induced nephrotoxicity, including N-acetylcysteine, anti-inflammatory drugs and antioxidant supplements, but none have an established role in patients being treated with cisplatin. Amifostine is the only FDA-approved agent for the reduction of cumulative renal toxicity in advanced ovarian and non–small-cell lung cancer patients receiving cisplatin [21]. However, use of this drug is limited by side effects (nausea, vomiting, hypotension). In addition, concerns about possible interference with the antitumor activity of cisplatin limits its use to clinical trials in tumors other than advanced ovarian and non–small-cell lung cancer patients.

Another strategy to prevent dehydration and cisplatin-induced nephrotoxicity, is to perform prophylactic percutaneous endoscopic gastrostomy (PEG) tube placement in those patients deemed at greatest risk of becoming malnourished or dehydrated during the course of treatment. The indication for prophylactic PEG placement is discussed in the multidisciplinary tumor board on a case-by case basis. Malnutrition, dysphagia and bilateral neck irradiation are among factors considered. In this retrospective study, 90 patients (73%) were treated with prophylactic PEG placement. During treatment, patients were monitored by a nutritionist, and if indicated a nasogastric feeding tube was placed in patients without PEG, or with PEG in the case of PEG-related complications or dysfunction. In this study, 20 patients (10 patients without PEG; 10 patients with PEG) were treated with (short-term) nasogastric feeding tube placement. Despite nastrogastric feeding tube placement, AKI occurred in 17 of these patients. Prophylactic PEG and feeding tube placement were not associated with a lower risk of AKI.

Reported predictors of cisplatin-induced AKI included older age and hypertension [22–24], female sex [22, 25], smoking, black ethnicity [22, 26], hypokalemia, hypoalbuminemia [23–25], concomitant use of other anticancer drugs, and single dose versus fractionated dose radiotherapy [27]. This retrospective study confirms the association of hypertension with cisplatin-induced AKI. No significant association with female sex was found, although both sexes were adequately represented in the study. The association of older age with AKI could not be confirmed, because elderly patients were underrepresented in this study (median age 60 years, range 30 to 74). Ethnicity could not be selected as a primary variable; included patients were predominantly white in this study. This also applied to serum albumin values, which were not measured in this study.

This study clearly demonstrates that CINV remains poorly controlled in a significant number of patients receiving CRT with high-dose cisplatin for LA-SSCHN, despite the use of guideline-consistent antiemetic therapy. Adherence to antiemetics in order to optimize CINV control for patients undergoing emetogenic chemotherapy is important, because adequate control of emesis prevents intravascular depletion of fluids and electrolytes, and therefore decreases the potential for cisplatin-induced nephrotoxicity. We have no data on adherence to antiemetics used in days 2 to 4, due to the retrospective design of this study. We were also unable to determine the severity of nausea or vomiting using an assessment tool in this retrospective study. Despite these limitations, there seems to be a clear need for further improvements in the management of CINV to minimize its negative impact. The benefit of olanzapine in controlling nausea and emesis has been suggested in previous trials, which showed that nausea and emesis were significantly reduced when olanzapine was added to guideline-directed prophylactic agents [28, 29]. This antiemetic regimen should be further explored in patients treated with CRT including high-dose cisplatin for LA-SCCHN.

Our results confirm previous observational studies’ findings that AKI is an independent risk factor for the development of chronic kidney disease [30, 31]. Decline of renal function was observed in both AKI and non-AKI patients at 3 and 12 months post-treatment. However, long-term decline in renal function was significantly more severe in AKI patients. In the current study, AKI did not have a negative impact both in terms of DFS and DSM. On the contrary, DSM and disease recurrence rates were numerically (but not statistically) higher in non-AKI patients*.* This could have several reasons. First, we did not have access to survival and disease recurrence data of all patients, which could have led to underreporting mortality and disease recurrence in the present study. Second, due to the retrospective nature of this study, patients were not stratified by prognostic risk factors, like primary tumor site, tumor stage, age or comorbidity at diagnosis, which may have resulted in unbalanced groups. Third, the follow-up period of 29 months was relatively short. Patients with AKI did not have inferior survival rates. In addition to the arguments already mentioned, this could also be explained by our data, showing that the majority of AKI and non-AKI patients (94%) received cisplatin with a cumulative dose of ≥200 mg/m^2^; only in 6% of patients cisplatin was discontinued after 1 cycle. Median cumulative dose of cisplatin was > 250 mg/m^2^ in both groups and not statistically different between treatment groups. This was well above the minimum dose of 200 mg/m^2^, which confers a survival benefit in LA-SCCHN patients treated with high-dose cisplatin-based concurrent CRT [32].

One of the strengths of our study was that associations between potential risk factors for AKI and outcome were studied in a well-characterized study population. AKI was also defined and graded according to KDIGO criteria, making it possible to identify low grade – but nevertheless clinically relevant – AKI. This study identifies a strong association between AKI and CINV, which is an important and potentially modifiable risk factor. Limitations were the single center retrospective nature of the study, and the relatively short follow up period of 2.5 years. Also, possible dose-response associations between the stage of AKI and outcome were not assessed. Finally, the effect of AKI and CINV on patients’ quality of life, and patients’ adherence to antiemetics could not be assessed due to the study’s retrospective design.

## Conclusions

AKI is a frequent complication of high-dose cisplatin-based CRT for patients with LA-SCCHN, despite adherence to guideline-consistent prevention therapy. CINV and hypertension are potentially modifiable and highly significant risk factors contributing to AKI. Studies investigating strategies to minimize AKI after high-dose cisplatin-based CRT for patients with LA-SCCHN are warranted.

## Data Availability

The datasets used and/or analysed during the current study are available from the corresponding author on reasonable request.

## Abbreviations

5-HT_3_ RA: Serotonin receptor antagonist
AKI: Acute kidney injury
CCI: Charlson comorbidity index
CI: Confidence interval
CINV: Chemotherapy-induced nausea and vomiting
CRT: Concurrent chemoradiation
CTCAE: Common toxicity criteria for adverse events
DFS: Disease free survival
DSM: Disease-specific mortality
ECOG: Eastern Cooperative Oncology Group
EMR: Electronic medical record
IQR: Interquartile range
KDIGO: Kidney disease improving global outcomes
LA-SCCHN: Locally advanced squamous cell carcinoma of the head and neck
NK_1_ RA: Neurokinin-1 receptor antagonist
Non-AKI: Non- acute kidney injury
OR: Odds ratio
SCr: Serum creatinine
SD: Standard deviation
